# Force Required in Mastication

**Published:** 1901-05

**Authors:** Frank M’S. Thomas


### FORCE REQUIRED IN MASTICATION.[1]

BY FRANK M'S. THOMAS.

THERE seems to be some confusion in the use of terms in the discussion of the forces used in mastication. The words *force, power,* and *work* are frequently used synonomously, and in order to a more correct understanding it is well to remember that *force* is that which produces, or tends to produce, a change in the motion of a body or mass; *work* is done when a force produces a displacement of a mass; *power* is the rate of doing work.

Let the elementary mechanics of the operation be considered. It is known that a force which may be applied directly upon the object to be moved (without the intervention of a lever system) produces a stress equal to the force. It is also known that a force producing motion does work, and that work is represented by the product of force (F) and the distance through which it is exerted (S); or, *work* $= F \times S$.

In this country work is usually represented in foot-pounds, or the product of the force in pounds and the distance in feet through which the force is exerted. It is seen that work embodies only force and distance. Power must now be considered. This is the work done in a certain time, in a unit of time. The unit of power is the "horse-power," which is a certain amount of work performed in one minute: $P = \frac{F \times S}{T}$.

The horse-power is the power required to raise thirty-three thousand pounds one foot high in one minute. It will be observed that power embodies force, distance, and time. It is also known that work has an equivalent in heat. The unit of heat is the

---

[1] At the recent discussion upon the views of Dr. Black, at the Academy of Stomatology, Philadelphia, there was such a variety of opinion expressed that the matter was referred to an electrical engineer to give a technical explanation of the probable force used in mastication.—EDITOR.

amount of heat required to raise the temperature of one pound of water one degree Farenheit. This is the B.T.U., or British thermal unit.

Experimentally it has been shown that one B.T.U. is equivalent to seven hundred and seventy-eight foot-pounds of work.

It is seen that the heat unit embodies simply a change of temperature of a certain weight or mass of substance. Now, as the horse-power is thirty-three thousand foot-pounds per minute, and as one foot-pound $= \frac{1}{778}$ B.T.U., we have: H.P. $= \frac{33000}{778} = 42.4$ B.T.U.'s per minute.

Keeping these units in mind, the action of mastication may be applied. Neglecting the lever-like structure of the jaw and the point of insertion of the muscles, we may assume that the total pressure exerted is one hundred pounds, to be exerted at one point. We may suppose the force to be one hundred pounds. Now, assuming that the jaws considered at this point move one inch before the teeth occlude, and no work is performed upon the substance masticated until the occluding teeth are one-quarter of an inch apart, and that the one hundred pounds is exerted uniformly over this distance (one-quarter inch), we would have one hundred pounds acting through one-forty-eighth of a foot, or 2.08, a little over two foot-pounds of work done by each occlusion of the teeth. This is a small amount of work.

Now, assume that the teeth occlude one hundred and twenty times per minute; what power is expended? $\frac{2.08 \times 120}{33000} =$ .00758 H.P., or less than one one-hundredth of a horse-power.

In remarks made in the discussion of Dr. Black's paper it was stated that a boy had cracked one hundred and fifty nuts in six minutes, requiring one hundred and fifty pounds to crush, and one hundred and fifty lemon-drops in six minutes, requiring sixty-five pounds to crush. It was pointed out that this aggregated many thousands of pounds, and this was regarded as a physiological impossibility. No mention was made of the distance through which these forces of one hundred and fifty and sixty-five pounds, respectively, were exerted. Thus the aggregate force in twelve minutes, without the distance being stated, is meaningless and misleading. Let this statement be taken and a value assumed for the unstated distance. Assuming, then, that when the nut or lemon-drop is distorted one-eighth of an inch it gives way and crushes or breaks. The teeth then only exert the maximum force through a distance of one-eighth of an inch. So that the work on each nut is $150 \times \frac{1}{8} \times \frac{1}{12} = 1.5$ foot-pounds, approximated. Now, in six minutes one hundred and fifty nuts were cracked; therefore, foot-pounds per minute $= \frac{1}{6} \times 1.5 \times 150 = 37.5$.

Now for the lemon-drops. $65 \times \frac{1}{8} \times \frac{1}{12} = .67$ of one foot-pound. In six minutes one hundred and fifty drops were cracked: foot-pounds per minute $= 67 \times \frac{1}{6} \times 150 = 16.7$.

The horse-power required to crack the nuts in this case would be H.P. $= \frac{37.5}{33000} = .0011$, or a little over one one-thousandth of a horse-power.

For the lemon-drops, $\frac{16.7}{33000} = .0005$, or about one two-thousandth of a horse-power.

This is a very small power, because while the force is considerable, the distance is very small. Of course, power is required to raise the jaw to bring it in contact with the nut, but this is too small to be important.

As a comparative example, it may be assumed that a man weighing one hundred and fifty pounds ascends a flight of steps, or a ladder ten feet high, and does this in ten seconds. The work is fifteen hundred foot-pounds, or eight thousand foot-pounds per minute.

The power $\frac{1500 \times 6}{33000} = 27$, or a little more than one-fourth of a horse-power. This is two hundred times the power of the nut-cracking example.

Upon this showing that mastication is a comparatively small portion of the work accomplished by the body, the writer sees nothing improbable in the values given by Dr. Black as to the forces exerted by the muscles of the mouth.

